# Identification of Nonylphenol and Glucolipid Metabolism-Related Proteins in the Serum of Type 2 Diabetes Patients

**Published:** 2019-12

**Authors:** Luo YA, Yu JIE, Yang MENGXUE, Wang PAN, Yang CHANGWEI, Xu JIE

**Affiliations:** 1. School of Public Health, Zunyi Medical University, Zunyi, Guizhou, P.R. China; 2. Department of Endocrinology, Affiliated Hospital of Zunyi Medical University, Zunyi, Guizhou, P.R. China; 3. Department of Nuclear Medicine, Affiliated Hospital of Zunyi Medical University, Zunyi, Guizhou, P.R. China

**Keywords:** Nonylphenol, T2 diabetes, PPAR-γ, SREBP-1c, Wnt5a

## Abstract

**Background:**

To identify serum nonylphenol (NP) and glucolipid metabolism-related proteins in Type 2 diabetes (T2D) patients.

**Methods:**

We performed a hospital-based, case-control study in patients admitted to the Department of Endocrinology, Hospital of Zunyi Medical University, Zunyi City, China from Mar to Nov of 2014. The study included 112 T2D cases diagnosed in accordance with the 2013 WHO Expert Committee Diabetes Diagnosing Criteria, and 125 healthy individuals with normal fasting blood glucose (FBG) when receiving physical examination in the same period in the Municipal Physical Examination Center. Blood samples from subjects in the 2 groups underwent detection of biochemical indices, including FBG, blood fat, and NP. Glucolipid metabolism-related proteins, including estrogen receptor (ER), sterol regulatory element-binding protein-1c (SREBP-1c), wingless-type MMTV integration site family member 5a (Wnt5a), and peroxisome proliferator-activated receptor-γ (PPAR-γ). These indices were compared between the 2 groups to analyze the correlation between serum NP levels and glucolipid metabolic proteins.

**Results:**

The subjects in the diabetes group had higher triglycerides (TG), total cholesterol (TC), NP, ER, SREBP-1c, Wnt5a, FBG, and TG levels than the healthy group, but lower levels of low-density lipoprotein cholesterol (LDL-C) and PPAR-γ than the healthy group. No significant differences in alanine aminotransferase (ALT) and aspartate aminotransferase (AST) were found between the two groups. The serum NP levels were shown to be positively correlated with SREBP-1c but negatively correlated with PPAR-γ.

**Conclusion:**

The serum NP levels of T2D patients is higher than the levels in healthy controls, and its levels correlate with SREBP-1c and PPAR-γ levels.

## Introduction

In accordance with WHO estimates in the 2016 Global Diabetes Report, about 0.422 billion people suffered from diabetes in 2014, and the incidence of diabetes across the world has almost doubled from 1980–2014, growing from 4.7% to 8.5%; most of the new cases appear in countries with low to moderate-income. According to research published in JAMA in Sep of 2013, in China, diabetes incidence has grown to 11.6%, and the population of diabetes patients has already reached 0.113 billion, not counting another 0.4934 billion potential cases (1, 2). The risk factors for diabetes remain quite complicated (3). In addition to genetic, pathological, physiological, social and psychological factors, and the mode of life, environmental factors also affect the incidence of this disease but are unfortunately often overlooked. Exposure to environmental endocrine-disrupting chemicals (EDCs) is one environmental risk factor. Among these EDCs, serum nonylphenol (NP) is a commonly encountered chemical that can enter the human body through several routes and disrupt the balance of metabolic hormones in vivo. NP is an intermediate chemical widely used in industrial processes, making it ubiquitous in daily life; it can be found in pesticides, lubricants, textile, foods, air, and in polluted soil and water. Such wide application has resulted in measureable levels of NP in both the plasma and urine of pregnant women, newborns, and pubertal students (4). It remains unclear as to what the acceptable range of NP is within the body, but the tolerable daily intake (TDI) of NP is 5 mg/kg bw/d for infants in Taiwan (5).

In a preliminary investigation, our research group found the lowest level of NP within the human body to be 404 ng/ml, while the highest was 622 ng/ml (6). We also found that diabetes patients had a higher in vivo load of NP when compared with healthy persons. In addition, according to animal studies, chronic exposure to NP may change the expression of genes and proteins related to glucolipid metabolism in such target organs like the pancreas, liver, and fat tissues (7–9).

Here, we attempted to confirm the levels of NP and glucolipid metabolism-related proteins in the serum and then analyze the correlation between these to identify potential risk factors for T2D. Our goal was to further define the mechanism(s) by which EDC exposure leads to hyperglycemia to identify potential future targets for reducing diabetes incidence and severity in clinical practice.

## Materials and Methods

### Subjects

All 112 cases in the diabetes group were diagnosed with T2D and were hospitalized in the Department of Endocrinology in the Affiliated Hospital of Zunyi Medical University in Zunyi City, Guizhou Province, China between Mar and Oct of 2014, after diagnosis in line with the 2013 WHO Expert Committee Diabetes Diagnosing Criteria. Those with diabetes caused by organic diseases concerning the central nervous system, endocrine system, and reproductive system were excluded from the study. In this group, there were 52 males and 60 females. Blood samples from 124 people in the healthy control group were all collected from 53 males and 71 females undergoing a physical examination in the hospital. Two groups did not significantly different in terms of age or sex.

### Sample collection

The subjects underwent 10 h overnight fast and denied taking of any drug 12 h before blood collection. Eight ml elbow vein blood was collected from subjects in a sitting position between 7:30 and 8:30 am; 5 ml was allocated to the coagulating tube for 30 min coagulation and then underwent serum separation. The serum-free blood was placed in an Eppendorf tube while serum was kept at −80 °C until further measurement of NP content, glucolipid metabolism-related proteins, and estrogen receptor. The rest remaining 3 ml of blood was put into an anticoagulant heparin tube for detecting the biochemical indicators like blood fat and fasting blood glucose (FBG). The biochemical detection was performed within 2 h.

All samples were acquired from two Grade A Class 3 hospitals with informed consent signed by the patients. The study passed Medical Ethics Committee review.

### Serum NP extraction and measurement.

Half a ml of serum was taken and added to 4 ml n-hexane-diethyl ether extractant (at volume ratio of 7:3). The mixture was placed in a vortex mixer for 30 sec mixing and then left still for 15 min. Supernatants were collected and dried in a 50 °C water bath and resolved with 0.5 ml acetonitrile before being put into the machine. The liquid chromatographic conditions were as follows: fluorescence detector with 275 nm excitation wavelength, 312 nm emission wavelength and mobile phase was composed of acetonitrile and 0.1% glacial acetic acid (at volume ratio of 85: 15), injected sample was 10 μl, and the flow rate was 1 ml/min.

### Biochemical indices detection

An automatic biochemical analyzer was used to detect FBG, alanine aminotransferase (ALT), aspartate aminotransferase (AST), triglyceride (TG), and cholesterol content. The operation was conducted as specified in the reagent kit instruction manual.

### Detection of PPAR-γ, Wnt5a, SREBP-1c, and ER

Glucolipid metabolism-related proteins were detected with an Enzyme Linked Immunosorbent Assay (ELISA). Metabolism-related proteins in serum were assessed by detecting the combination of antibody and antigen.

## Results

The study included 112 diabetes patients ages 48.6±10.3-yr-old and 125 people ages 44.0±14.0-yr-old with normal fasting blood glucose. The 105 male subjects were 47.3±12.3-yr-old on average, while the 131 female subjects were 45.2±12.8-yrold on average. There were no significant differences in the two groups in terms of age or sex. The diabetes group had significantly higher FBG and NP values than the healthy group (Table 1).

**Table 1:** Clinical and laboratory characteristics about the study subjects (n=236

***Variable***	***Healthy group***	***Diabetes group***	**P*-value***
Gender (Male/Female)	53/71	51/61	0.079
Age(yr)	50.18±9.95	49.13±9.27	0.290
FPG	5.09±0.6	11.0±4.30^*^	0.000
NP	515.7±65.0	605.2±127.6 ^*^	0.000
ALT(U/L)	30.48±16.94	25.89±19.87	0.232
AST(U/L)	26.49±8.62	24.13±13.74	0.892
TG(mmol/L)	1.61±0.93	2.31±1.43 ^*^	0.000
TC(mmol/L)	4.89±0.90	4.93±1.04^*^	0.042
HDL-C(mmol/L)	1.2±0.27	1.1±0.26	0.924
LDL-C (mmol/L)	3.1±0.68	3.0±0.97^*^	0.030
PPAR-γ(mmol/L)	25.0±13.8	20.0±9.7^*^	0.004
Wnt5a (pg/ml)	18.3±4.4	18.1±9.4^*^	0.000
SREBP-1c (ng/ml)	13.8±3.3	16.1±6.6 ^*^	0.000
ER(ng/L)	26.6±10.5	28.3±8.2 ^*^	0.002

Compared to the healthy group, the diabetes group demonstrated significant increases in TG and TC, while the LDL-C in the diabetes group was lower than in the healthy group, and there were no significant differences in ALT, AST, or HDL-C between the two groups (Table 1).

The diabetes group had significantly lower PPAR-γ and Wnt5a levels than the healthy group, but had higher SREBP-1cand ER levels (Table 1).

There was a positive correlation between NP and SREBP-1c and NP and ER, but a negative correlation between NP and PPAR-γ. There was no significant correlation between NP and Wnt5a (Table 2).

**Table 2:** Correlation coefficient between NP and PPAR-γ, Wnt5a, SREBP-1cand ER

	***PPAR-γ***	***Wnt5a***	***SREBP-1c***	***ER***
r	−0.220	0.064	0.522	0.330
P	0.001	0.240	0.000	0.000

Logistic regression analysis showed that the variables (biochemical indexes, age, NP) were independently associated with PPAR-γ, SREBP-1c, and ER (Table 3).

**Table 3:** Logistic enter regression showing variables independently associated with PPAR-γ, SREBP-1c, and ER

***Dependent variable***	***C***	***B***	***SE***	***β***	***t***	***P***
PPAR-γ	NP	−0.034	0.006	−0.302	−5.926	<0.001
	TG	6.040	0.513	0.612	11.764	<0.001
	LDL-C	−0.854	2.356	−0.019	−0.363	0.717
	Age	0.001	0.049	0.001	−0.028	0.978
SREBP-1c	NP	0.029	0.002	0.594	11.839	<0.001
	TG	−1.622	0.217	−0.382	−7.460	<0.001
	LDL-C	1.974	0.998	0.100	1.979	0.049
	Age	0.060	0.021	0.144	2.865	0.005
ER	NP	0.0308	0.005	0.350	5.686	<0.001
	TG	−0.681	0.481	−0.089	−1.416	0.158
	LDL-C	−0.933	2.208	−0.026	−0.122	0.673
	Age	0.164	0.046	0.218	3.546	<0.001

## Discussion

PPAR-γ, a member of the nuclear receptor family, is field related to lipogenesis regulation and plays a vital role in adipocyte differentiation regulation and the metabolism of sugar, fat, and energy. It can control fat storage and release, maintain in vivo energy balance, and regulate the stability of insulin and blood glucose (10). Here, we found that PPAR-γ expression was reduced, while the NP content was elevated in the serum of diabetes patients. In addition, the NP content was negatively correlated with PPAR-γ levels, caused by exposure to NP alone or the joint effects of NP and other substances, such that PPAR-γ expression was affected during in vivo glycometabolism regulation, resulting in increased blood glucose levels.

There are three isomers of sterol regulatory element binding proteins (SREBPs): SREBP-1a, SREBP-1c, and SREBP-2 (11–13). In humans, SREBP-1c is mainly distributed in the liver and fat cells. It is also referred to as an adipocyte determination and differentiation factor, because on one hand it acts as the main regulator for fatty acid synthesis and expression of genes related to glucose metabolism, and on the other hand, it is an important nuclear transcription factor. Regulated by multiple hormones and nutrients, such as insulin, leptin, glucagon, and liver X activated receptor, SREBP-1c mediates insulin-based regulation of the expression of several genes, while it participates in B cell dysfunction genes is and development, and plays a vital role in the pathogenesis of insulin resistance, obesity, and T2D. The lipid synthesis of hepatic cells is regulated by SREBP-1c (14–18). Therefore, overexpression of SREBP-1c may promote fatty acid synthesis in hepatic cells, such that triglycerides (TGs) and fatty acids gather in those cells to raise the TG serum levels. Here, we identified a correlation between serum NP content and SREBP-1c concentration, which was statistically significant. In addition, regression analysis found that SREBP-1c was affected by NP, TG, and HDL-C. Therefore, SREBP-1c appears to regulate the metabolism of fatty acid and glucose to raise FBG and TGs in the serum and enable NP to enter into the body to upregulate the expression of SREBP-1c through certain interference effects. The consequence is hyperglycemia and hyperlipidemia. Furthermore, SREBP-1c and TC may interact with each other to form a vicious cycle in which NP and age are primary instigating factors for the development of T2D.

Wnt5a is a member of the Wnt family, members of involved lipogenesis (19,20). Wnt5a can cause abnormal adipocyte metabolism through some nonclassical pathways. Newly diagnosed T2D patients had higher serum Wnt5a levels, which was consistent with the findings of this paper. However, no correlation was detected between NP and Wnt5a. Nonetheless, there was a significant correlation between NP and estrogen receptor (ER), with NP and age acting as key factors influencing ER. NP exerted an estrogen-like toxic effect, in alignment with its predicted role.

The research indicates: 1) that serum NP levels tend to be higher in diabetic patients, 2) NP may inhibit the expression of PPAR-γ by itself or jointly with other substances, which in turn interferes with the ability of insulin to regulate blood glucose, 3) NP promotes overexpression of SREBP-1c to impair the metabolic regulation of fatty acids and glucose, which causes hyperglycemia, and 4) no correlation was detected between Wnt5a and NP.

## Conclusion

We found a close relationship between SREBP-1c and PPAR-γ and NP-induced hyperglycemia. However, a larger patient population is required to make firm conclusions. Our future plans include a larger study where we will examine the effects of multiple EDCs so as to further investigate the correlation between NP and diabetes.

## Ethical considerations

Ethical issues (Including plagiarism, informed consent, misconduct, data fabrication and/or falsification, double publication and/or submission, redundancy, etc.) have been completely observed by the authors.
